# Systematic review and meta‐analysis of the efficacy and safety of [^177^ Lu]Lu‐edotreotide ([^177^ Lu]Lu‐DOTATOC) for the treatment of neuroendocrine tumors

**DOI:** 10.1111/jne.70103

**Published:** 2025-11-08

**Authors:** Richard P. Baum, Julia G. Fricke, Tristan Ruhwedel, Holger Amthauer, Erika Patricia Azorin‐Vega, Dieter Hörsch, Riccardo Laudicella, Vikalp Maheshwari, Martin A. Walter, Berna Degirmenci Polack, Simon F. Spiegl, Guillaume P. Nicolas

**Affiliations:** ^1^ Advanced Theranostics Center for Radiomolecular Precision Oncology, Deutsche Klinik für Diagnostik (DKD HELIOS Klinik) Curanosticum Wiesbaden‐Frankfurt Wiesbaden Germany; ^2^ Department of Theragnostics, Division of Nuclear Medicine, ENETS Centre of Excellence (CoE) University Hospital Basel Basel Switzerland; ^3^ Department of Nuclear Medicine Charité—Universitätsmedizin Berlin, Corporate Member of the Free University of Berlin and of Humboldt University of Berlin Berlin Germany; ^4^ Department of Radioactive Materials Instituto Nacional de Investigaciones Nucleares (ININ) State of Mexico Mexico; ^5^ Zentralklinik Bad Berka GmbH Bad Berka Germany; ^6^ Department of Biomedical and Dental Sciences and Morpho‐Functional Imaging Nuclear Medicine, University of Messina Messina Italy; ^7^ Parexel International Hyderabad Telangana India; ^8^ Institute of Nuclear Medicine, University Hospital Basel Basel Switzerland; ^9^ ITM SE Munich Germany

**Keywords:** [^177^Lu]Lu‐edotreotide, neuroendocrine tumor (NET), peptide receptor radionuclide therapy (PRRT), radiopharmaceutical therapy (RPT)

## Abstract

[^177^Lu]Lu‐edotreotide is a radiopharmaceutical therapy (RPT) targeting somatostatin receptors, which are commonly overexpressed on neuroendocrine tumors (NETs). This systematic literature review and meta‐analysis describes the efficacy and safety of [^177^Lu]Lu‐edotreotide in patients with NETs. To date, there has been no meta‐analysis of data for this specific RPT. PubMed, EMBASE, Cochrane databases, and abstracts from select congresses were searched for eligible studies (February/October 2024). Meta‐analysis was performed using fixed and random‐effects models. The primary objective was to evaluate the efficacy of [^177^Lu]Lu‐edotreotide in terms of objective response rate (ORR; complete + partial response) in the subgroup of patients with gastro‐enteropancreatic NETs (GEP‐NETs) and those with any NETs, irrespective of origin (All‐NETs). Secondary outcomes included disease control rate (DCR; best overall response of complete response + partial response + stable disease), median progression‐free survival (mPFS), and median overall survival (mOS). Unpublished/updated data were requested from the investigators of the included publications where needed to provide missing information/enable evaluation of additional outcomes. Safety/tolerability data for [^177^Lu]Lu‐edotreotide were also reviewed. Eight eligible studies were identified for inclusion in the meta‐analysis, all in the advanced disease setting (5/8 included patients with progressive NETs). Most patients had grade 1/2 NETs (grade 1: 11%–63%; 2: 30%–79%; 3: 4%–11%). Updated data were provided for four of these studies. Overall, ORR and DCR were reported in six studies (GEP‐NETs, *n* = 222; All‐NETs, *n* = 423), mPFS in five studies (GEP‐NETs, *n* = 294; All‐NETs, *n* = 267), and mOS in six studies (GEP‐NETs, *n* = 256; All‐NETs, *n* = 408). Meta‐analysis revealed consistently high heterogeneity (*I* ^2^ >70%) across outcomes/patient populations. Patients with GEP‐NETs appeared to have better outcomes than those with All‐NETs in terms of ORR (34% vs. 19%), DCR (78% vs. 57%), mPFS (24.9 vs. 18.6 months), and mOS (44.8 vs. 39.1 months), respectively. Safety/tolerability data were inconsistently reported, but grade 3/4 toxicities were rarely noted during [^177^Lu]Lu‐edotreotide treatment. These results support the effectiveness and safety of [^177^Lu]Lu‐edotreotide as a treatment for patients with advanced NETs and suggest a potentially more favorable prognosis for those with GEP‐NETs than for the broader All‐NETs population. However, these results should be interpreted with caution due to the high level of heterogeneity. Encouraging ORRs and high DCRs were noted, indicating that [^177^Lu]Lu‐edotreotide effectively stabilized disease in most patients. Although safety/tolerability data were inconsistently published across studies, [^177^Lu]Lu‐edotreotide was generally well tolerated. Overall, these findings suggest that the efficacy and safety of [^177^Lu]Lu‐edotreotide are in line with those reported for other RPTs in similar clinical settings.

**Clinical Trial Registration**: PROSPERO 2024 CRD42024518028.

## 1 INTRODUCTION

Neuroendocrine neoplasms are a rare and diverse group of tumors, around 80%–90% of which are classified as the more indolent neuroendocrine tumors (NETs). NETs can occur in many different organs, with the gastrointestinal tract (62%–67%) and lung (22%–27%) being the most frequent sites of origin.^1^, ^2^, ^3^ The incidence and prevalence of NETs are generally rising globally. Incidence rates for gastro‐enteropancreatic (GEP) neuroendocrine neoplasms vary geographically; a rate of 6.1 cases per 100,000 was recently reported for the United States^4^ and, for Europe, rates ranged from 2.5 cases per 100,000 in Germany to 8.35 cases per 100,000 in Norway.^5^ Because many patients with NETs are diagnosed late,^6^ they often present with distant metastases, especially to the liver.^7^, ^8^ At this point, curative resection may not be possible and systemic treatment approaches are required.

NETs commonly over‐express somatostatin receptors (SSTRs), making them amenable to treatment with radioactively labeled somatostatin analogs (SSAs), known as radiopharmaceutical therapy (RPT, or peptide receptor radionuclide therapy [PRRT]). RPT has emerged as an exciting and rapidly evolving new treatment option for patients with well‐differentiated NETs.^6^, ^9^ Among the most widely used agents in RPT are the SSTR‐targeted, somatostatin‐modified peptide fragments DOTA‐[Tyr^3^]‐octreotide (DOTATOC or edotreotide) or DOTA‐[Tyr^3^, Thr^8^]‐octreotate (DOTATATE). Both of these agents are conjugated via a chelator (DOTA), which allows labeling with β‐emitting radiometals such as yttrium‐90 (^90^Y), lutetium‐177 (^177^Lu), or other suitable radionuclides such as gallium‐68 (^68^Ga).^10^ Both agents target SSTR, but their affinity profiles differ for each SSTR subtype, and affinity is also affected by the radiometal they are chelated to.^11^ For example, as well as having high affinity for SSTR2, [^68^Ga]Ga‐labeled edotreotide binds SSTR5 with ~5‐fold higher affinity than [^68^Ga]Ga‐labeled DOTATATE.^11^, ^12^ Conversely, the in vitro affinity of [^68^Ga]Ga‐labeled DOTATATE in binding SSTR2 is ~10‐fold higher than that of [^68^Ga]Ga‐labeled edotreotide, which may impact their effectiveness in the functional imaging of NETs with a predominance of SSTR2 such as GEP‐NETs.^13^ Although radiolabeled DOTATATE and edotreotide are used in both imaging and treatment, the high SSTR affinity of ^68^Ga‐labeled DOTA‐1‐NaI^3^‐octreotide ([^68^Ga]Ga‐DOTANOC) correlated with high uptake in normal organs and a high whole‐body dose,^14^ so this agent is only used diagnostically.^15^ There are also significant differences in the pharmacokinetic and normal organ dosimetry profiles of [^177^Lu]Lu‐DOTATATE and [^177^Lu]Lu‐edotreotide ([^177^Lu]Lu‐DOTATOC), with edotreotide‐based agents appearing to have the lowest uptake or dose delivered in normal tissue and/or a better tumor‐to‐kidney ratio overall, compared with DOTATATE‐based agents in some studies.^13^, ^14^, ^16^ However, findings have been conflicting in this area, with a further study reporting an improved tumor‐to‐kidney ratio for [^177^Lu]Lu‐DOTATATE, based on longer tumor and kidney residence times compared with [^177^Lu]Lu‐edotreotide.^17^ In this study, higher tumor doses could be achieved with [^177^Lu]Lu‐DOTATATE, despite reaching the maximum tolerated kidney dose. Ongoing substudies from the phase 3 COMPETE trial will further evaluate the dosimetry profile of [^177^Lu]Lu‐edotreotide.

Despite the need for practical considerations arising from the gamma emissions that are also emitted by this radionuclide,^18^ Lutetium‐177‐radiolabeled drugs are now the clinical standard in RPT.^9^ [^177^Lu]Lu‐DOTATATE is indicated in the United States^19^ and Europe^20^ for the treatment of certain patients with SSTR‐positive GEP‐NETs; phase 3 trials are ongoing for [^177^Lu]Lu‐edotreotide.

Given the rarity of NETs and the relatively new field of RPT, systematic literature reviews (SLRs) and/or meta‐analyses are particularly useful in aiding the understanding of treatment effects. Several recent SLRs and/or meta‐analyses have evaluated outcomes in patients with NETs treated with RPT,^21^, ^22^, ^23^, ^24^, ^25^, ^26^ showing promising results. However, all but one of these only reported on the efficacy of [^177^Lu]Lu‐DOTATATE and/or [^90^Y]Y‐DOTATOC; none have focused specifically on [^177^Lu]Lu‐edotreotide. The objective of the current study was to describe the efficacy and safety of [^177^Lu]Lu‐edotreotide in patients with NETs through an SLR and meta‐analysis, using broad inclusion criteria. To date, there has been no meta‐analysis of data for this specific RPT.

## 2 METHODS

This study was conducted in line with the Preferred Items for Systematic Review and Meta‐Analyses (PRISMA) 2020 statement^27^ (ID: PROSPERO 2024 CRD42024518028).

### 2.1 Eligibility criteria

Full eligibility criteria for this analysis are shown in Table S1. In brief, any studies including individuals of any age or sex with any type of NET (including GEP‐NETs) who received RPT with [^177^Lu]Lu‐edotreotide alone (coadministration of SSAs that were not radioactively labeled was permitted) and that reported efficacy or safety outcomes of interest were eligible for inclusion.

### 2.2 Data sources

PubMed, EMBASE, and Cochrane databases were searched on February 13, 2024, for eligible studies (published in English, with no date restrictions). In addition, abstracts from 2020 to 2024 from the European Neuroendocrine Tumor Society (ENETS) congress were manually searched (all other relevant congresses were covered by EMBASE). The PubMed search was re‐run on October 23, 2024, to check for additional new publications of interest before conducting the analyses. Search terms included “177 Lu edotreotide,” “177 Lu‐DOTATOC,” “DOTA‐Phe1‐Tyr3‐octreotide,” and other drug synonyms. Full details of the search terms and strings that were used in this study are included in Table S2.

### 2.3 Study selection and data extraction

Study records were exported from online literature databases to Microsoft Excel for study selection and decision recording. In the first stage of the process, after excluding duplicate records, titles and abstracts were screened by two reviewers independently to select records for further full‐text evaluation to establish eligibility. Study investigators were contacted for additional information/data to establish eligibility, as necessary. Researchers were blinded to each other's decisions until screening was complete; any differences were resolved by consensus. Reference lists of eligible publications and of reviews published since 2020 were also examined to identify any additional relevant studies.

Relevant data were extracted into an Excel database from study documents by one reviewer, then the extraction was checked by a second reviewer. Hereby, the following data were extracted (where available): study information, including design, phase, timelines, follow‐up duration, [^177^Lu]Lu‐edotreotide activity per cycle, cumulative activity (GBq), number of cycles administered and the time interval between cycles, number of patients, and method of response assessment (Response Evaluation Criteria In Solid Tumors [RECIST], World Health Organization [WHO], etc.). Information on patient age, sex, clinical setting (e.g., line of treatment, previous treatments), NET type/grade, and distribution of metastasis at baseline was also collated. Efficacy items included best overall tumor response (complete response [CR], partial response [PR], stable disease [SD], progressive disease [PD]), objective response rate (ORR), disease control rate (DCR), progression‐free survival (PFS), and overall survival (OS). The frequency and severity of adverse events (AEs; those of interest to this review were hematologic toxicities, renal/hepatic toxicities, and secondary malignancies) were also summarized, where available.

Unpublished and/or updated information/data were requested from the investigators of the included publications where needed, to provide missing information and/or enable evaluation of additional outcomes. Additional and/or updated data were provided and included in this analysis from the following investigators: Dr. Laudicella,^28^ Prof. Walter,^29^ Dr. Nicolas,^30^ and Dr. Ruhwedel.^31^, ^32^ Where relevant, the latest updated data rather than the original published data is reported for these studies. The updated dataset from Ruhwedel et al. has since been published and is now cited throughout, where appropriate.^33^

### 2.4 Outcomes and prioritization

Data were analyzed in two populations of patients receiving treatment with [^177^Lu]Lu‐edotreotide: the subgroup of patients specifically with GEP‐NETs and the more heterogeneous “All‐NETs” population (all patients with NETs, irrespective of origin). The primary outcome for the meta‐analysis was ORR (proportion of patients with a CR or PR). Secondary outcomes analyzed in the meta‐analysis were: DCR (proportion of patients with a best overall tumor response of a CR, PR, or SD [irrespective of duration]), best overall tumor response (CR, PR, SD, PD), PFS (median; months), and OS (median; months).

The following safety/tolerability outcomes were summarized (but not analyzed), where available: proportions of patients with AEs (type and severity grade [e.g., as assessed via Common Toxicity Criteria for Adverse Events]), hematologic toxicities (e.g., anemia, thrombocytopenia, lymphocytopenia, and neutropenia, by grade), renal/hepatic toxicities (by grade), and the incidence of secondary malignancies, such as myelodysplastic syndrome (MDS) and acute myeloid leukemia (AML).

### 2.5 Quality of evidence and risk of bias

The studies shortlisted for analysis were assessed for risk of bias using validated quality assessment tools. All observational studies, including prospective and retrospective designs, were assessed using the Newcastle‐Ottawa Scale (Table S3). Detailed results of this critical appraisal are presented in Table S4. Two reviewers independently conducted quality assessments while blinded to each other's evaluations. Any discrepancies were resolved by consensus discussion, or, when necessary, adjudicated by a third reviewer. Publication bias assessment was not conducted as the analysis included fewer than 10 eligible studies, which falls below the methodologically recommended minimum threshold for reliable publication bias detection. The interpretation of findings was based on the formal risk of bias assessments that were conducted and considered within the context of the available literature in this therapeutic area.

### 2.6 Data synthesis

#### 2.6.1 Feasibility assessment

A feasibility assessment was undertaken to assess the suitability of conducting a meta‐analysis of data from the identified studies. The standards of various health technology assessment bodies were followed—for example, the recommendations of the National Institute for Health and Care Excellence (NICE) technical support documents 2 and 3 on the conduct of meta‐analysis.^34^, ^35^ The studies identified in the SLR were assessed for heterogeneity in trial design and study characteristics (e.g., inclusion/exclusion criteria, blinding status, treatment strategies [cycles of therapy, dose regimen, etc.]), and patient/clinical characteristics (age, ethnicity, performance status, etc.). Kaplan–Meier methodology employing the log hazard approach was utilized to estimate the median OS/PFS. This methodology was applied specifically in cases where patient‐level data were accessible. Microsoft Excel and R software (Version 4.1.1) were used for the feasibility assessment.

#### 2.6.2 Meta‐analysis

Meta‐analysis was undertaken using fixed (using Mantel–Haenszel methods^36^) and random‐effects (using DerSimonian and Laird methods^37^) models. Inverse‐variance weighted meta‐analysis of proportions was performed using the Freeman–Tukey double‐arcsine transformation method.^38^ Results were assessed for both clinical diversity and methodological diversity. Assessments of statistical heterogeneity were undertaken by examining the forest plots and results of the *I* ^2^ statistic, which indicate the presence of statistical heterogeneity (*I* ^2^ value of ≥25%: low heterogeneity; ≥50%: moderate heterogeneity; ≥75%: considerable heterogeneity).^39^ Sensitivity analyses were performed using alternate transformations (log and logit) for DCR and ORR outcomes. These were not performed for continuous outcomes such as median OS and median PFS.

## 3 RESULTS

### 3.1 Search results and study characteristics

**FIGURE 1:**
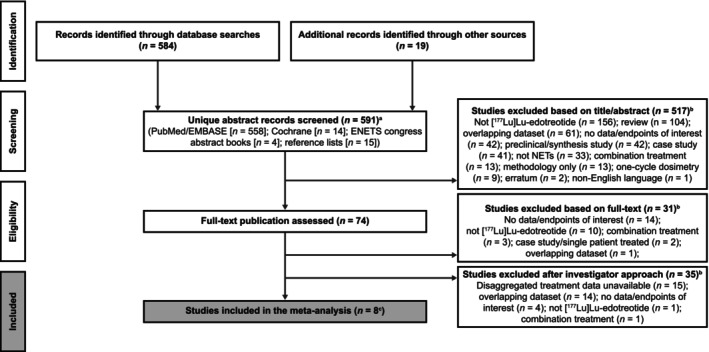
PRISMA diagram. ^a^The PubMed search was repeated on October 23, 2024, to check for any relevant new articles that had been published since the original searches were conducted on February 13, 2024; the results were reviewed and no relevant new studies were identified. ^b^Many publications were excluded for multiple reasons; therefore, just one key reason for exclusion is listed for the purpose of this chart. ^c^Updated and/or additional data were provided by some study investigators. ENETS, European Neuroendocrine Tumor Society.

We screened the titles and abstracts of the 591 unique publications identified in the preliminary searches, then screened the full text of 74 of these articles, resulting in the identification of eight relevant publications for inclusion in the meta‐analysis (Figure 1).^8^, ^28^, ^29^, ^30^, ^31^, ^40^, ^41^, ^42^ Data/information from two other publications reporting additional data from the Ruhwedel et al. study^32^, ^33^ was included in some efficacy analyses, where appropriate.

These eight publications comprised one retrospective, non‐comparative, phase 2 study/analysis,^40^ two prospective feasibility/translational studies,^41^, ^42^ and five other retrospective analyses,^8^, ^28^, ^29^, ^30^, ^31^ one of which utilized data from three prospective studies^29^ (Table 1). In general, inclusion/eligibility criteria were similar across these eight studies, although there were some differences in study entry requirements/assessments (Table S5). All studies included patients with a confirmed diagnosis of advanced/metastatic NETs, and five of the eight studies specifically included patients with PD. Geographically, four of the studies were conducted in Switzerland (Basel), two in Germany (Berlin and Bad Berka), and one each in Mexico and Italy. Study timelines varied considerably, spanning from 1997 to 2019.

**TABLE 1:** Study information.

	Forrer et al. 2005,^41^, ^a^	Theiler et al. 2021,^30^, ^a^	Kobayashi et al. 2021^8^	Radojewski et al. 2015,^26^, ^a^	Ruhwedel et al. 2021,^31^, ^33^, ^a^	Baum et al. 2016^40^	Luna‐Gutiérrez et al. 2023^42^	Laudicella et al. 2022,^28^, ^a^
Study design/phase	Prospective feasibility study	Retrospective matched cohort study	Retrospective study	Retrospective pooled analysis of three prospective studies	Retrospective study	Retrospective phase 2 study	Prospective translational study	Retrospective study
Number of patients^b^	27	51	19	141	141	56	187	38
NET population(s) reported	All‐NET	All‐NET GEP‐NET	All‐NET	All‐NET	All‐NET GEP‐NET	All‐NET GEP‐NET	All‐NET GEP‐NET	GEP‐NET
[^177^Lu]Lu‐edotreotide dosage and schedule								
Per‐cycle activity, GBq (range)	7.4	7.4	5.5 (5.5–7.4)^c^	7.4	7.4	7.0 (3.5–10.0)^c^	7.4	5.5 (3.5–7.4)^c^
Treatment interval	N/A	10–12‐weeks	≥6 weeks	≥6 weeks	10–12 weeks	3–4^c^ months	8–10 weeks	9.2 [±4.2]^d^ weeks
Median cycles administered, *n* (range)	1^e^	NR (2–4)	3 (1–3)	2 (1–5)	3 (1–6)	2.1 (1–4)^d^	NR (2–8)	5 (5–7)
Median cumulative activity, GBq (range)	7.4^e^	NR	NR	13.5 [±6.5]^d^	NR	13.1 (3.5–29.2)	NR	29 [±1.5]^d^
Median follow‐up, months (range)	11.0 [±4.0]^d^ (4–17)	NR	29.9^f^ (4.8–93.1)	11.3^f^ (1.0–157.6)	19^g^ (15.8–25.0)	16.1 [±12.4]^d^	36.0^h^ [±16]	14.5 (1.2–74.4)
Response criteria/assessment method	WHO	Other^i^	RECIST 1.1	RECIST	RECIST 1.1	RECIST 1.1	SPECT or PET/CT^j^	SSTR PET/CT

Abbreviations: CT, computed tomography; (GEP‐)NET, (gastro‐enteropancreatic) neuroendocrine tumor; IQR, interquartile range; N/A, not applicable; NR, not reported; OS, overall survival; PET, positron emission tomography; RECIST, Response Evaluation Criteria In Solid Tumors; SPECT, single photon emission computed tomography; SSTR, somatostatin receptor; WHO, World Health Organization.

^a^ Some updated and/or additional data/information included as provided by the investigator in this study.

^b^ Number of patients with NETs who received [^177^Lu]Lu‐edotreotide treatment alone and were included in this analysis.

^c^ Median (range) value.

^d^ Mean value [±SD].

^e^ Single fixed‐activity study.

^f^ Overall median follow‐up, irrespective of treatment received (data not reported for [^177^Lu]Lu‐edotreotide radiopharmaceutical therapy alone).

^g^ Median (IQR) follow‐up for patients without disease progression/relapse (median follow‐up for patients who had not died was 54.3 [IQR: 41.0–74.3] months).

^h^ Median follow‐up for the patients included at the January 2019 cut‐off date (*n* = 81; follow‐up was 52 months at the time of the OS analysis).

^i^ Physician assessment based on all available clinical, biochemical, and imaging data.

^j^ Alongside patient clinical and biochemical data.

Five of the eight studies reporting per‐cycle activity information used a [^177^Lu]Lu‐edotreotide 7.4/7.45 GBq,^29^, ^30^, ^31^, ^41^, ^42^ and all seven studies reporting treatment‐scheduling information administered [^177^Lu]Lu‐edotreotide activities at ≥6‐week intervals (typically 10–12 weeks; maximum interval ~17 weeks)^8^, ^28^, ^29^, ^30^, ^31^, ^40^, ^42^ (Table 1). Five studies reported the median number of cycles administered; patients received a median of two or three cycles of [^177^Lu]Lu‐edotreotide treatment in four of these (range: 1–8),^8^, ^29^, ^31^, ^40^ while a median of five cycles was administered in the remaining study.^28^ In one study, patients were administered a single fixed activity of 7.4 GBq (1 cycle).^41^ Only three studies reported average cumulative [^177^Lu]Lu‐edotreotide activity administered (mean/median of ~13 GBq [range: 3.5–29.2] in two studies^29^, ^40^ and mean of 29 GBq in the remaining study^28^). The higher cumulative activity reported in the Laudicella et al. study was achieved despite using a lower median per‐cycle activity (5.5 GBq), as patients received a higher median number of treatment cycles.^28^ Although not reported in the remaining studies, average cumulative activity would be expected to be closer to the upper end of the range reported above, based on the activity per cycle and number of cycles administered (Table 1).

Where reported, average overall follow‐up ranged from 11 to 36 months.^8^, ^28^, ^29^, ^31^, ^40^, ^41^, ^42^ Assessment of treatment response varied between studies, with RECIST version 1.1 being the most frequently used criteria^8^, ^31^, ^40^ (Table 1). The data sources and analysis populations used for these analyses are shown in Table S6.

### 3.2 Patient characteristics

Updated study data from the investigators were included for four of these studies,^28^, ^29^, ^30^, ^33^ resulting in data from a maximum of 294 patients with GEP‐NETs and 489 patients with All‐NETs available for inclusion, with numbers varying slightly by analysis, depending on what data/outcomes were available in each study.

Baseline patient demographics and disease characteristics for the eight included studies are shown in Table 2. Mean and/or median age was between 58 and 64 years (overall range: 58–80 years) in seven of the eight studies.^8^, ^28^, ^29^, ^32^, ^40^, ^41^, ^42^ The remaining study was a matched cohort study, comparing safety and efficacy of RPT in elderly (aged ≥79 years) versus disease‐matched younger patients (aged 60–70 years), and so the overall study population had a higher mean age of 80 years.^30^ Proportionally, more male than female patients were included in the majority (seven of eight) of the studies.^8^, ^28^, ^29^, ^30^, ^32^, ^40^, ^41^ All studies reported on the type of NETs^8^, ^28^, ^29^, ^30^, ^32^, ^40^, ^41^, ^42^; most patients had tumors of gastro‐entero (26%–58%) or pancreatic (18%–53%) origin. Data on NET grade were reported in six studies^8^, ^28^, ^30^, ^32^, ^40^, ^42^; overall, most patients had grade 1 (11%–63%) or 2 (30%–79%) NETs. Few patients with grade 3 NETs were included (4%–11% overall). Seven studies reported metastatic site location^8^, ^28^, ^29^, ^30^, ^32^, ^40^, ^42^; liver metastases were most common and were present in ≥50% of patients in these studies. Information on the prior therapy types was available from all eight studies^8^, ^28^, ^29^, ^30^, ^32^, ^40^, ^41^, ^42^; overall, many patients had received prior treatment with SSAs (37%–100%) and many had also undergone prior surgery (20%–82%). Information on the number of prior lines of treatment was available in four studies^28^, ^30^, ^32^, ^42^; most patients had received 1 (2%–42%) or 2 (31%–98%) prior lines of treatment.

**TABLE 2:** Baseline patient demographics and characteristics in the included studies.

	Forrer et al. 2005^41^ (*n* = 27)	Theiler et al. 2021^30^ (*n* = 51)^a^	Kobayashi et al. 2021^8^ (*n* = 19)	Radojewski et al. 2015^29^ (*n* = 141)	Ruhwedel et al. 2021/Wetz et al. 2023^31^, ^32^ (*n* = 141)^b^	Baum et al. 2016^40^ (*n* = 56)	Luna‐Gutiérrez et al. 2023^42^ (*n* = 187)^a^	Laudicella et al. 2022^28^ (*n* = 38)^c^
Age, median, years	58^d^	80	62	62	64	64^d^	63	58
Sex, female, *n* (%)	10 (37)	20 (39)	7 (37)	59 (42)	52 (37)	27 (48)	105 (56)	15 (40)
Type of NET, *n* (%)								
Gastro‐entero	11 (41)	29 (57)	5 (26)	61 (43)	80 (57)	28 (50)	105 (56)	22 (58)^a^
Pancreatic	11 (41)	13 (25)	10 (53)	25 (18)	38 (27)	15 (27)	50 (27)	16 (42)^a^
Pulmonal	1 (4)	5 (10)	–	–	10 (7)	–	17 (9)	–
Other	4 (15)	4 (8)	4 (21)	54 (38)	13 (9)	13 (23)	15 (8)	–
NET grade, *n* (%)								
1	–	32 (63)	2 (11)	–	30 (21)	19 (34)	47 (44)^e^	8 (21)
2	–	19 (37)	15 (79)	–	100 (71)	23 (41)	32 (30)^e^	28 (74)
3	–	–	2 (11)	–	5 (4)	3 (5)	–	2 (5)
Unknown	–	–		–	6 (4)	11 (20)	28 (26)^e^	–
Site(s) of metastases,^f^ *n* (%)								
Liver/hepatic	–	43 (84)	18 (95)	71 (50)	135 (96)	38 (68)	110 (59)	32 (84)
Bone/osseous	–	15 (29)	6 (32)	23 (16)	54 (38)	15 (27)	41 (22)	12 (32)
Lymph node	–	29 (57)	11 (58)	–	118 (84)	37 (66)	21 (11)	26 (68)
Lung	–	–	1 (5)	–	6 (4)	7 (13)	–	1 (3)
Peritoneum	–	10 (20)	–	–	24 (17)	15 (27)	7 (4)	4 (11)
Other	–	6 (12)	–	–	–	5 (9)	–	22 (58)
Prior treatment type,^g^ *n* (%)								
SSAs	10 (37)	27 (53)	19 (100)	–	108 (77)	24 (43)	183 (98)	31 (82)
Surgery	17 (63)	25 (49)	9 (47)	79 (56)	93 (66)	46 (82)	37 (20)	21 (55)
Chemotherapy	6 (22)	7 (14)	6 (33)	17 (12)	41 (29)	10 (18)	–	12 (32)
Radiotherapy	–	2 (4)	–	47 (33)	5 (4)	–	–	1 (3)
Prior treatment lines, *n* (%)								
None	–	7 (14)	–	–	6 (4)	–	–	–
1	–	18 (35)	–	–	32 (23)	–	2 (2)^e^	16 (42)
2	–	16 (31)	^h^	–	68 (48)	–	104 (98)^e^	14 (37)
≥3	–	10 (20)	–	–	35 (25)	–	–	8 (21)

Abbreviations: (GEP‐)NET, (gastro‐enteropancreatic) neuroendocrine tumor; SSA, somatostatin analog.

^a^ Data calculated using updated information provided by the investigator, where available.

^b^ Data taken from Wetz et al. 2023.^32^

^c^ All patients in this study had GEP‐NETs.

^d^ Mean value reported.

^e^ Data available for 106 patients.

^f^ Patients could have had more than one site of metastases.

^g^ Patients could have had more than one prior treatment type and/or other treatment.

^h^ Patients received a median of two (range: 0–8) prior treatments (including surgical resection).

### 3.3 Meta‐analysis of efficacy

This meta‐analysis revealed consistently high heterogeneity (*I* ^2^ >70%) across outcomes and patient subgroups, indicating significant inter‐study variability in treatment effects, patient characteristics, or methodologies. As this level of heterogeneity could not be explained on the basis of the content of the original publications, results from the more conservative random‐effects model approach were prioritized, so that the uncertainty of the single‐effect estimates was reflected in wider confidence intervals (CIs). The results of the sensitivity analysis were similar to the base‐case results for all outcomes (Table S7), indicating that the meta‐analysis results were robust.

#### 3.3.1 Treatment response

Response to treatment was assessed via RECIST 1.1 in three studies,^8^, ^33^, ^40^ via RECIST in one study,^29^ and via WHO criteria in another^41^ (Table 1). The remaining studies did not use specific response‐assessment criteria but assessed treatment response using more real‐world methods of SSTR positron emission tomography (PET)/computed tomography (CT),^28^ SSTR single photon emission CT (SPECT)/CT,^42^ or a combination of approaches.^30^

**FIGURE 2:**
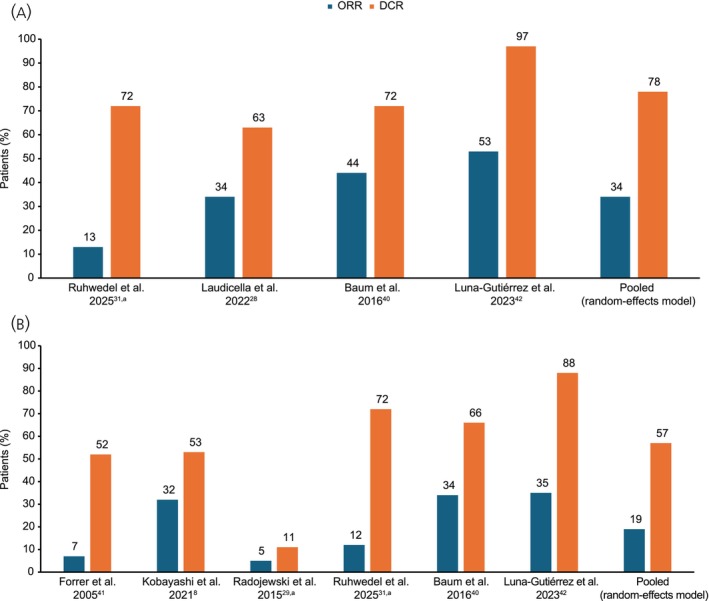
ORRs and DCRs, by study and pooled, in the (A) GEP‐NET and (B) All‐NET populations. ^a^Updated and/or additional unpublished data provided by the investigator of this study. DCR, disease control rate; (GEP‐)NET, (gastro‐enteropancreatic) neuroendocrine tumor; ORR, objective response rate.

Seven studies reported treatment response data during [^177^Lu]Lu‐edotreotide treatment,^8^, ^28^, ^29^, ^33^, ^40^, ^41^, ^42^ of which four reported data for patients with GEP‐NETs^28^, ^33^, ^40^, ^42^ and six reported data for patients with All‐NETs.^8^, ^29^, ^33^, ^40^, ^41^, ^42^ A total of 222 patients with GEP‐NETs were included in the response analysis, and 65 of these patients had an objective response (53 PR, 12 CR) (Figure 2A). Of the 423 patients with All‐NETs included in this analysis, 69 had an objective response (57 PR, 12 CR) during [^177^Lu]Lu‐edotreotide treatment (Figure 2B). In these studies, reported ORRs ranged between 13%–53% and 5%–35% in patients with GEP‐NETs and All‐NETs, respectively. After double‐arcsine transformation to stabilize variance in the presence of heterogeneity (random‐effects model), the overall calculated ORR was 34% (95% CI: 17–54) in patients with GEP‐NETs and 19% (95% CI: 8–32) in patients with All‐NETs. Notably, one study^33^ in the GEP‐NETs analysis had a markedly lower ORR than in the remainder.^28^, ^40^, ^42^ Notably, patients in this study received salvage treatment comprising only a single cycle of [^177^Lu]Lu‐edotreotide (7.4 GBq).^33^ Likewise, the ORRs in two studies in the All‐NETs analysis^29^, ^41^ were markedly lower than in the remaining studies.^8^, ^33^, ^40^, ^42^

A total of 222 patients with GEP‐NETs and 423 patients with All‐NETs were included in the analysis of DCR. In the GEP‐NETs population, a total of 166 patients had disease control (65 with an objective response, 101 with SD). In the All‐NETs population, a total of 215 patients had disease control (69 with an objective response, 146 with SD). The crude DCRs from these studies ranged between 63%–97% and 11%–88% for patients with GEP‐NETs and All‐NETs, respectively. The calculated DCR (random‐effects model) was 78% (95% CI: 60–92) in those with GEP‐NETs (Figure S1A) and 57% (95% CI: 33–79) in patients with All‐NETs (Figure S1B). Of note, the DCR in one of the studies in the All‐NET analysis^29^ was markedly lower than in the remaining studies.^8^, ^33^, ^40^, ^41^, ^42^

#### 3.3.2 PFS

**FIGURE 3:**
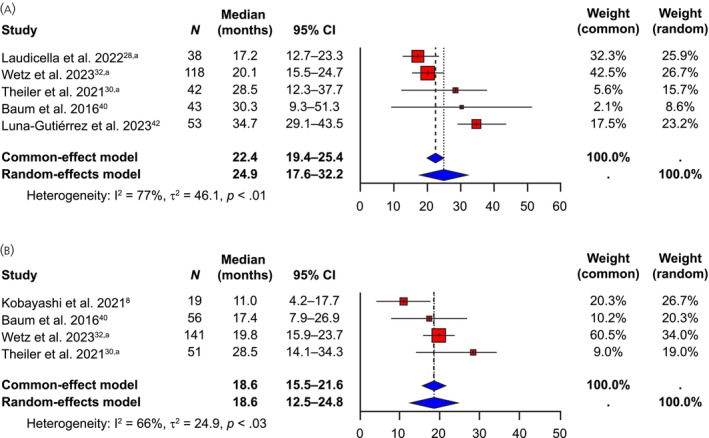
Median PFS (months) in the (A) GEP‐NET and (B) All‐NET populations. ^a^Updated and/or additional unpublished data provided by the investigator of this study. (GEP‐)NET, (gastro‐enteropancreatic) neuroendocrine tumor; PFS, progression‐free survival.

Five studies (encompassing a total of 294 patients) reported median PFS data in patients with GEP‐NETs during [^177^Lu]Lu‐edotreotide treatment; medians ranged from 17.2 to 34.7 months.^28^, ^30^, ^32^, ^40^, ^42^ Median PFS was digitized from the reported Kaplan–Meier curve in one study and included only progression events^42^; the remaining studies included both progression and death events.^28^, ^30^, ^32^, ^40^ The pooled median PFS estimate was 24.9 (95% CI: 17.6–32.2) months in this patient population (random‐effects model) (Figure 3A).

Four studies (encompassing a total of 267 patients) reported median PFS data in patients with All‐NETs during [^177^Lu]Lu‐edotreotide treatment; medians ranged from 11.0 to 28.5 months.^8^, ^30^, ^32^, ^40^ In one study, it was unclear if the reported PFS data included death events^8^; the remainder included both progression and death events.^30^, ^32^, ^40^ The random‐effects model generated a pooled median PFS estimate of 18.6 (95% CI: 12.5–24.8) months in this patient population (Figure 3B).

#### 3.3.3 OS

**FIGURE 4:**
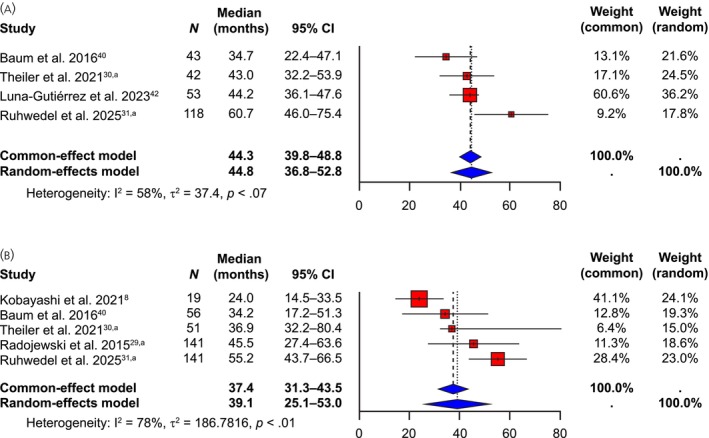
Median OS (months) in the (A) GEP‐NET and (B) All‐NET populations. ^a^Updated and/or additional unpublished data provided by the investigator of this study. (GEP‐)NET, (gastro‐enteropancreatic) neuroendocrine tumor; OS, overall survival.

Four studies (encompassing a total of 256 patients) reported median OS data for patients with GEP‐NETs; medians ranged from 34.7 to 60.7 months.^30^, ^33^, ^40^, ^42^ The random‐effects model generated a pooled median OS estimate of 44.8 (95% CI: 36.8–52.8) months in this patient population (Figure 4A). Where available, average follow‐up in these studies varied between 14.5 and 36.0 months.^28^, ^33^, ^40^, ^42^

Five studies (encompassing a total of 408 patients) reported median OS data in patients with All‐NETs during [^177^Lu]Lu‐edotreotide treatment; medians ranged from 24.0 to 55.2 months.^8^, ^29^, ^30^, ^33^, ^40^ The random‐effects model generated a pooled median OS estimate of 39.1 (95% CI: 25.1–53.0) months in this patient population (Figure 4B). Where available, average follow‐up for OS in these studies varied between 11.3 and 52 months.^8^, ^29^, ^33^, ^40^, ^42^

### 3.4 Safety and tolerability

Excluding RPT combination treatment, six of the studies reported safety/tolerability data for [^177^Lu]Lu‐edotreotide RPT alone that were of interest to this review.^8^, ^29^, ^30^, ^40^, ^41^, ^42^ However, the different types of safety/tolerability data were not reported consistently across studies. Only one study reported the overall proportion of patients experiencing any AEs (61%^40^); no serious AEs occurred in this study. The incidence of serious AEs was not mentioned in any of the remaining studies; discontinuations due to AEs were not mentioned in any of the studies. The frequency of AEs of interest to this review was reported in six studies^8^, ^29^, ^30^, ^40^, ^41^, ^42^ (Table 3). Four studies reported data on individual hematologic AEs.^8^, ^29^, ^40^, ^41^ Grade 1/2 anemia (21%–51%^8^, ^29^, ^40^, ^41^), thrombocytopenia (23%–37%^8^, ^29^), leukopenia (14%–26%^8^, ^29^), neutropenia (26%^8^), and lymphocytopenia (47%^8^) were commonly reported. Severe hematologic AEs were rare (grade 3/4 anemia: 4%^40^, ^41^; thrombocytopenia: 1%^29^; and lymphocytopenia: 16%^8^). Grade 3/4 neutropenia, thrombocytopenia, or lymphocytopenia occurred in a total of 4% of patients in a further study.^42^ In the remaining study, hematologic and renal AEs were classified as subacute (occurring within 12 weeks of a cycle) or long‐term (occurring within 1 year of RPT).^30^ Subacute hematologic toxicity of grade ≥3 or with an increase of ≥2 grades occurred in 25% of patients overall; long‐term hematologic toxicity occurred in 11% of patients overall.^30^

**TABLE 3:** AEs of interest (per our study protocol), by severity grade (Common Toxicity Criteria for Adverse Events, where stated).

	Forrer et al. 2005^41^ (*n* = 27)	Kobayashi et al. 2021^8^ (*n* = 19)	Radojewski et al. 2015^29^ (*n* = 141)	Baum et al. 2016^40^ (*n* = 56)
Anemia, *n* (%)				
Grade 1 or 2	9 (33)	8 (42)^a^	72 (51)	7 (21)^b^
Grade 3 or 4	1 (4)	0	0	1 (4)^c^
Thrombocytopenia, *n* (%)				
Grade 1 or 2	–	7 (37)^a^	32 (23)	–
Grade 3 or 4	–	0	2 (1)	–
Leukopenia, *n* (%)				
Any grade/grade 1 or 2	–	5 (26)^a^	20 (14)	–
Grade 3 or 4	–	0	0	–
Neutropenia, *n* (%)				
Grade 1 or 2	–	5 (26)^a^	–	–
Grade 3 or 4	–	0	–	–
Lymphocytopenia, *n* (%)				
Grade 1 or 2	–	9 (47)^d^	–	–
Grade 3 or 4	–	3 (16)	–	–
Renal toxicity/dysfunction, *n* (%)				
Grade 1 or 2	–	5 (26)^a^	–	–
Grade 3 or 4	–	0	13 (10)^e^	–
Hepatic dysfunction (AST), *n* (%)				
Grade 1 or 2	–	7 (37)^a^	–	–
Grade 3 or 4	–	0	–	–
Hepatic dysfunction (ALT), *n* (%)				
Grade 1 or 2	–	4 (21)^f^	–	–
Grade 3 or 4	–	1 (5)	–	–

Abbreviations: AE, adverse event; ALT, alanine aminotransferase; AST, aspartate transaminase.

^a^ Reported as “any/all” grade, but no grade 3/4 events occurred, so all events are assumed to be grade 1/2.

^b^ Of 33 patients with no anemia at baseline, seven developed grade 1 anemia during treatment (two additional patients progressed from grade 1 at baseline to grade 2; calculated from percentages reported in the paper).

^c^ Of 23 patients with grade 1 or 2 anemia at baseline, one progressed to grade 3 during treatment (calculated from percentages reported in the paper).

^d^ Reported as “any/all” grade, three grade 3/4 events subtracted to give assumed grade 1/2 rate.

^e^ Percentage calculated on the basis of event data reported in the paper, assuming each event occurred in a separate patient (13 severe renal toxicity events occurred in the 130 patients with available data in the [^177^Lu]Lu‐edotreotide group).

^f^ Reported as “any/all” grade, one grade 3/4 event subtracted to give assumed grade 1/2 rate.

Rates of renal dysfunction/toxicity during [^177^Lu]Lu‐edotreotide treatment were reported in three studies^8^, ^29^, ^30^; grade 1/2 events occurred in 26% of patients,^8^ with 10% having severe renal AEs in another study.^29^ In the Theiler et al. study,^30^ subacute and long‐term renal toxicities of grade ≥3 occurred in 6% and 8% of patients overall, respectively. One study reported on hepatic dysfunction^8^; grade 1/2 dysfunction based on altered aspartate aminotransferase and alanine aminotransferase levels occurred in 37% and 21% of patients, respectively. Another study stated there was no hepatotoxicity or correlation between renal function and the use of [^177^Lu]Lu‐edotreotide indicative of moderate/severe renal toxicity.^42^ Grade 3/4 dysfunction occurred in 5% of patients (based on alanine aminotransferase alterations).^8^ No cases of MDS or AML were reported in those receiving RPT with [^177^Lu]Lu‐edotreotide alone.

## 4 DISCUSSION

To our knowledge, this is the first SLR and meta‐analysis to specifically address the efficacy and safety of [^177^Lu]Lu‐edotreotide in patients with NETs. The reported data were all in the advanced disease setting, with most patients having grade 1/2 NETs, PD at baseline, and receiving second‐line [^177^Lu]Lu‐edotreotide treatment. Meta‐analysis of the available data suggested that ORRs (34% vs. 19%), DCRs (78% vs. 57%), and median PFS (24.9 vs. 18.6 months) and OS (44.8 vs. 39.1 months) outcomes were higher in patients with GEP‐NETs than in those with All‐NETs, respectively. Nonetheless, CIs were wide, and a high level of heterogeneity was consistently noted across these analyses, especially in the All‐NET population; therefore, these results should be interpreted with caution. The noted variations in outcomes could be due to differences in study methodologies or patient characteristics and underscore the complexity of the management of patients with NETs.

Safety and tolerability data were reported inconsistently across the identified studies, but, in general, suggest that [^177^Lu]Lu‐edotreotide treatment is well tolerated and rarely associated with severe hematologic or hepatic events. Low rates of grade ≥3 renal events were also observed, as expected, now that a protective solution of arginine‐lysine is routinely administered during RPT. Furthermore, no cases of MDS or AML were reported during RPT with [^177^Lu]Lu‐edotreotide alone. In general, these findings are consistent with the data reported for [^177^Lu]Lu‐DOTATATE in a recent review, in which ~4%–11% of patients receiving this agent were reported to have experienced grade 3/4 hematologic toxicity, with acute nephrotoxicity or hepatic toxicity noted in ~1%–5% and ~1%–4% of patients, respectively.^43^ However, ~1%–2% and ~0.7%–1.1% of patients have been reported to develop MDS and leukemia following [^177^Lu]Lu‐DOTATATE treatment.

Although the results of this analysis suggest that patients with GEP‐NETs have better outcomes than those with All‐NETs during [^177^Lu]Lu‐edotreotide treatment, the data were insufficiently granular to permit evaluation of efficacy by NET tumor origin. Limited data were reported in some of the publications included in the meta‐analysis, but findings differed between studies. For example, in the Kobayashi et al. study, there was no significant difference in median PFS between patients with pancreatic versus gastro‐entero versus other NETs (12.2 vs. 14.1 vs. 11.0 months, respectively; *p* =.414); however, data were reported only for the group overall, including those receiving [^177^Lu]Lu‐edotreotide plus [^90^Y]Y‐DOTATOC.^8^ In the multivariable Cox model reported by Ruhwedel et al., the overall effect of primary tumor location was not statistically significant (*p* = .11), but there was a nominally significant association for shorter OS in patients with pancreatic versus gastrointestinal NETs during [^177^Lu]Lu‐edotreotide therapy (*p* = .04).^33^ However, there are prognostic differences between NETs of different origin, depending on the aggressiveness of disease.^44^, ^45^

Overall, our findings for [^177^Lu]Lu‐edotreotide are in line with results from other recent SLRs/meta‐analyses in this area.^21^, ^22^, ^23^, ^24^, ^26^, ^43^ However, only one of these meta‐analyses included [^177^Lu]Lu‐edotreotide data,^23^ and just two of the 22 included studies evaluated [^177^Lu]Lu‐edotreotide (results were not reported separately for this agent). Nonetheless, the pooled ORRs (25%–35%) and DCRs (79%–83%) reported in that analysis were in line with our findings. Of note, an SLR protocol with aims similar to ours was registered on PROSPERO in November 2022 but is not yet published.^46^ However, that review only considers studies in which [^177^Lu]Lu‐edotreotide or [^90^Y]Y‐DOTATOC was compared with at least one other treatment; our review took a broader approach, including any study in which patients with NETs were treated with [^177^Lu]Lu‐edotreotide, with or without a comparator group. We also considered a broader range of safety/tolerability outcomes, including hematologic and renal/hepatic toxicities.

Efficacy data in patients with NETs have been reported for [^177^Lu]Lu‐DOTATATE plus SSA versus high‐dose SSA alone in the phase 3 NETTER‐1 and NETTER‐2 trials. In the primary analysis of NETTER‐1, [^177^Lu]Lu‐DOTATATE plus SSA was associated with an ORR of 18% in patients with locally advanced unresectable or metastatic midgut NETs that had progressed on prior SSA therapy.^7^ Prior RPT was not permitted in this study, and the median PFS was not reached in the [^177^Lu]Lu‐DOTATATE group but was significantly improved versus control treatment (median PFS: 8.4 months). In the final analysis, the median OS for [^177^Lu]Lu‐DOTATATE plus SSA was 48.0 months versus 36.3 months for the control group (no significant difference between treatments).^47^ In the first‐line NETTER‐2 trial, which included patients with advanced grade 2/3 GEP‐NETs, the ORR reported for [^177^Lu]Lu‐DOTATATE plus SSA was 43%, and the median PFS was 22.8 months.^48^ However, indirect comparisons of our findings with NETTER‐1 and NETTER‐2 data may be inaccurate/misleading due to differences in study design, patient populations, and methodology. Nonetheless, our results are consistent with those from an observational study of [^177^Lu]Lu‐DOTATATE in patients with predominantly metastatic GEP‐NETs.^49^ Here, the overall ORR was 39% (SD was found in a further 43% of patients), and median PFS and OS were 29 and 63 months, respectively. The best ORR (53%) and OS (median: 71 months) outcomes were seen in patients with pancreatic NETs.^49^

After completion of the present meta‐analysis, primary analysis results from the COMPETE trial became available. This phase 3 trial compares [^177^Lu]Lu‐edotreotide versus everolimus in patients with advanced grade 1/2 GEP‐NETs, mostly in the second‐line setting.^50^ In COMPETE, patients received [^177^Lu]Lu‐edotreotide every 12 weeks for a maximum of 4 cycles (7.5 GBq/cycle). The study design and population in COMPETE are more comparable with those of the present study, and our meta‐analysis results for the GEP‐NETs population are very similar to those reported in this trial. For example, in COMPETE, the median PFS during [^177^Lu]Lu‐edotreotide treatment was 23.9 months (vs. 24.9 months here)—a significant improvement versus the comparator (14.1 months; HR: 0.68 [95% CI: 0.48–0.95]; *p* = .025).^50^ Interestingly, the median (interim) OS was 63.4 months in the [^177^Lu]Lu‐edotreotide group^51^—higher than that found in the present meta‐analysis of “real‐world” studies (44.8 months).

It remains to be seen whether the possible differences in SSTR2 binding affinity, organ dosimetry, and pharmacokinetic profile between [^177^Lu]Lu‐DOTATATE and [^177^Lu]Lu‐edotreotide translate to clinical benefits for either agent.

We acknowledge that there are limitations to our meta‐analysis. There was a high level of heterogeneity across outcomes and patient subgroups, indicating significant inter‐study variability in treatment effects, patient characteristics, and/or methodologies. For example, there were differences in the RPT treatment protocols used in the eight studies, including fixed versus variable dosing, the number of cycles administered, and total cumulative activity, underscoring a lack of standardization in [^177^Lu]Lu‐edotreotide administration. This variability may have contributed to the differences in treatment outcomes observed across studies. For instance, a lower ORR was observed for the All‐NETs population in the single‐dose study^41^; based on the per‐cycle activity and number of cycles administered, the lower ORR and DCR rates in the Radojewski et al. study^29^ could also be related to a lower administered activity, although cumulative activity was not specifically reported. Furthermore, differences in how treatment response was assessed/evaluated could explain some of the observed variability in ORRs and DCRs across studies. DCR was calculated purely as the sum of observed CRs, PRs, and SDs (irrespective of duration/timeframe), so is most relevant for those patients with PD at baseline. Although most of the patients included in the meta‐analysis received second‐line [^177^Lu]Lu‐edotreotide treatment, some heterogeneity in the level of pretreatment remained, which could also have impacted the observed efficacy. In some studies, patients were treated with multiple lines of therapy (sometimes including other RPTs^41^) before [^177^Lu]Lu‐edotreotide treatment.^8^, ^28^, ^30^, ^32^

Much of the heterogeneity observed here could stem from the fact that the studies included in this meta‐analysis spanned a broad inclusion period (1997–2019), and so represent the continual evolution in imaging and treatment practices over this time. For example, there has been a switch from [^111^Indium]‐octreotide planar/SPECT imaging to [^68^Ga]‐SSTR PET/CT and a shift from fixed, single‐cycle RPT dosing without renal protection to multi‐cycle regimens, guided where possible by dosimetry information. Such time‐dependent changes systematically distort response and toxicity outcomes and therefore challenge the comparability of pooled outcomes.

Despite these methodological differences, the available data allowed for a robust comparison of patient populations, providing valuable insights into the demographic and clinical profiles of patients with NETs treated with [^177^Lu]Lu‐edotreotide. Four of the studies included some patients with well‐differentiated grade 3 tumors (all ≤11%^8^, ^28^, ^33^, ^40^); however, efficacy was not reported separately for this patient subgroup. Therefore, further data are required to clarify the efficacy of [^177^Lu]Lu‐edotreotide in patients with grade 3 NETs to support the promising data available in the literature on the efficacy/safety of RPT in this setting.^52^

[^177^Lu]Lu‐edotreotide continues to be evaluated in an ongoing clinical trial program in patients with NETs. The phase 3 COMPOSE trial (NCT04919226) compares the efficacy and safety of [^177^Lu]Lu‐edotreotide with physician's choice standard‐of‐care chemotherapy as a first–/second‐line treatment in patients with aggressive, SSTR‐positive grade 2/3 GEP‐NETs. In the COMPOSE trial, patients will receive up to 6 cycles of [^177^Lu]Lu‐edotreotide, given at 6‐ to 8‐week intervals^53^ at 7.5 GBq/cycle. An additional phase 3, investigator‐sponsored trial (LEVEL; NCT05918302) is evaluating [^177^Lu]Lu‐edotreotide versus everolimus in treatment‐naïve and treatment‐experienced patients with SSTR‐positive lung and thymus NETs, while the phase 1 KinLET trial (NCT06441331) is assessing [^177^Lu]Lu‐edotreotide for the treatment of pediatric patients with recurrent, progressive, or refractory SSTR‐positive tumors. The results of these studies should help further clarify the role of [^177^Lu]Lu‐edotreotide in the treatment of patients with NETs.

## 5 CONCLUSION

The results of this SLR and meta‐analysis support the effectiveness of [^177^Lu]Lu‐edotreotide as a treatment for patients with advanced NETs, especially tumors of gastrointestinal or pancreatic origin. [^177^Lu]Lu‐edotreotide treatment was associated with favorable ORRs and high DCRs in this setting and was generally well tolerated. Although safety/tolerability data were not published consistently across studies, grade 3/4 hematologic, renal, or hepatic AEs were rarely reported. Overall, the data described here suggest that the efficacy and safety of [^177^Lu]Lu‐edotreotide are consistent with those reported for other RPTs in similar clinical settings.

## AUTHOR CONTRIBUTIONS

**Richard P. Baum:** Conceptualization; methodology; writing – review and editing; data curation; investigation; supervision; validation. **Julia G. Fricke:** Conceptualization; methodology; data curation; investigation; validation; writing – review and editing. **Tristan Ruhwedel:** Conceptualization; methodology; data curation; investigation; validation; writing – review and editing. **Holger Amthauer:** Conceptualization; methodology; data curation; investigation; validation; writing – review and editing. **Erika Patricia Azorin‐Vega:** Conceptualization; methodology; data curation; investigation; validation; writing – review and editing. **Dieter Hörsch:** Conceptualization; methodology; data curation; investigation; validation; writing – review and editing. **Riccardo Laudicella:** Conceptualization; methodology; data curation; investigation; validation; writing – review and editing. **Vikalp Maheshwari:** Methodology; data curation; investigation; validation; writing – review and editing; formal analysis. **Martin A. Walter:** Conceptualization; methodology; data curation; investigation; validation; writing – review and editing. **Berna Degirmenci Polack:** Conceptualization; methodology; data curation; investigation; validation; writing – review and editing. **Simon F. Spiegl:** Supervision; project administration; writing – review and editing; resources. **Guillaume P. Nicolas:** Conceptualization; methodology; data curation; investigation; validation; formal analysis; supervision; visualization; writing – review and editing.

## CONFLICT OF INTEREST STATEMENT

Richard P. Baum has acted as an advisor/consultant for 3B Pharmaceuticals, Full Life Technologies, and ITM SE. He has received honoraria from Monrol and Novartis and has stock/stock options in ITM SE and Telix Pharmaceuticals. Tristan Ruhwedel has received travel support from Novartis. Holger Amthauer has received grants to the institute from ITM for participation in the COMPETE and COMPOSE clinical trials, has acted as an advisor/speaker for Novartis, Pfizer, and Sirtex, and has received travel support from Sirtex. Julia G. Fricke, Erika Patricia Azorin‐Vega, Dieter Hörsch, Riccardo Laudicella, and Martin A. Walter have no competing interests to declare. Vikalp Maheshwari is an employee of Parexel International, which received funding support from ITM Oncologics to perform the meta‐analysis. Berna Degirmenci Polack and Simon F. Spiegl are employees of ITM Oncologics. Guillaume P. Nicolas has acted as an advisor/speaker for Ipsen, ITM, Janssen Cilag, Novartis, Sanofi, and Siemens Healthineers.

## Supporting information


**Data S1.** Supporting Information.

## Data Availability

The data included in this systematic literature review and meta‐analysis is taken directly from the associated published articles (the authors provided updated information, where stated in the manuscript; data may be available on request to the responsible author).
