# Supplementary material for: Systematic review and meta‐analysis of the efficacy and safety of [177 Lu]Lu‐edotreotide ([177 Lu]Lu‐DOTATOC) for the treatment of neuroendocrine tumors
Source: J Neuroendocrinol. 2025 Nov 8;38(1):e70103. doi: 10.1111/jne.70103 (PMC12799327; doi:10.1111/jne.70103)
Supplement: Supplementary file 1 — Data S1. Supporting Information. [file JNE-38-e70103-s001.docx]

**Supplemental material**

**SUPPLEMENTAL TABLE 1** Study eligibility criteria.

|  | **Inclusion criteria** | **Exclusion criteria** |
| --- | --- | --- |
| Population | Individuals of any age or sex with NETs of any type |  |
| Intervention | [^177^Lu]Lu-edotreotide | [^177^Lu]Lu-edotreotide used in combination with other anticancer treatments, including other radioligand therapies (except for SSAs that were not radioactively labeled, amino acids, and antiemetic agents) |
| Comparison | Any or none |  |
| Outcomes | Efficacy and/or safety (as detailed in the outcomes section) |  |
| Type of study | Randomized controlled trials,  non-randomized controlled trials, quasi-experimental studies, prospective and retrospective studies, cohort studies, and case series (if patients are analyzed, or can be analyzed as a group) | Case studies, review articles, news, editorials |
| Context | Studies from any clinical setting | Preclinical, pharmacokinetic, and pharmacodynamic studies |
| Geography | No restriction |  |
| Language | English language only |  |
| Publication date and status | No restriction |  |

Abbreviations: NET, neuroendocrine tumor; SSA, somatostatin analog.

**SUPPLEMENTAL TABLE 2** Search strings and terms used in the study,
by information source.

| **PubMed^a^** | ("lutetium"[all fields] OR "lutetium"[Mesh] OR "lutetium Lu 177"[all fields] OR "177Lu lutetium"[all fields] OR "177Lu"[all fields] OR "177 Lu"[all fields]) AND (("dotatoc"[all fields] OR "dota-toc"[All fields] OR "DOTA-Phe1-Tyr3-octreotide"[all fields] OR "DOTA Phe1 Tyr3 octreotide"[all fields]) OR ("edotreotide"[all fields] OR "edotreotide"[MESH])) OR ("lutetium Lu 177 edotreotide"[All fields] OR "Lu 177 edotreotide"[All fields] OR "177Lu edotreotide"[All fields] OR "177 Lu edotreotide"[All fields] OR "Lu-DOTATOC"[All fields] OR "177Lu-DOTATOC"[All fields] OR "[177Lu]Lu-DOTA-TOC"[All fields]) |
| --- | --- |
| **EMBASE** | (('lutetium':ab,ti OR 'lutetium lu 177':ab,ti OR '177lu lutetium':ab,ti OR '177lu':ab,ti OR '177 lu':ab,ti) AND ('dotatoc':ab,ti OR 'dota-toc':ab,ti OR 'dota-phe1-tyr3-octreotide':ab,ti OR 'dota phe1 tyr3 octreotide':ab,ti)) OR 'edotreotide':ab,ti OR 'lutetium lu 177 edotreotide':ab,ti OR 'lu 177 edotreotide':ab,ti OR '177lu edotreotide':ab,ti OR '177 lu edotreotide':ab,ti OR 'lu-dotatoc':ab,ti OR '177lu-dotatoc':ab,ti OR '[177lu]lu-dota-toc':ab,ti OR '(177)lu-dotatoc' OR 'edotreotide lutetium lu-1772' OR 'dotatoc lu-177' OR '(177ludota)-toc' OR 'edotreotide lutetium lu-177' OR rgo812q0c8 OR 'lutetium lu177 edotreotide' OR 'lutetium lu-177 dota-tyr3-octreotide' OR 'unii-rgo812q0c8' OR 'lutetium lu 177-edotreotide' OR 'lutetium lu-177-edotreotide' OR 'who 12604' OR '321835 55 6' OR 'lutetium lu-177 edotreotide' OR 'lutetium (177lu) edotreotide' OR '(s2,s7-cyclo[[4,7,10-tris(carboxylato-ko-methyl)-1,4,7,10-tetraaza-k3n4,n7,n10-cyclodec-1-yl]acetyl-ko]-d-phenylalanyl-l-cysteinyl-l-tyrosyl-d-tryptophyl-l-lysyl-l-threonyl-l-cysteinyl-l-threoninol)lutetium[177lu](3-)' OR '[n2.1-([4,7,10-tris[(carboxylato-kappao)methyl]-1,4,7,10-tetraazacyclododecan-1-yl-kappa4n1,n4,n7,n10]acetyl-kappao)- s3.2,s3.7-cyclo(d-phenylalanyl-l-cysteinyl-l-tyrosyl-d- tryptophyl-l-lysyl-l-threonyl-l-cysteinyl-l- threoninol)](177lu)lutetium' OR 'lutetium-177lu, [n-[2-[4,7,10-tris[(carboxy-o)methyl]-1,4,7,10-tetraazacyclododec-1-yl]acetyl-o]-d-phenylalanyl-l-cysteinyl-l-tyrosyl-d-tryptophyl-l-lysyl-l-threonyl-n-[(1r,2r)-2-hydroxy-1-(hydroxymethyl)propyl]-l-cysteinamide cyclic (2-->7)-disulfidato(3-)]' |
| **Cochrane databases** | ("lutetium" OR "lutetium Lu 177" OR "177Lu lutetium" OR "177Lu" OR "177 Lu") AND (("dotatoc" OR "dota-toc" OR "DOTA-Phe1-Tyr3-octreotide" OR "DOTA Phe1 Tyr3 octreotide") OR ("edotreotide")) OR ("lutetium Lu 177 edotreotide" OR "Lu 177 edotreotide" OR "177Lu edotreotide" OR "177 Lu edotreotide" OR "Lu-DOTATOC" OR "177Lu-DOTATOC" OR "[177Lu]Lu-DOTA-TOC") |
| **ENETS congress abstracts (2020–2024)** | The following search terms were used to manually search for relevant congress abstracts:  ("lutetium" OR "lutetium Lu 177" OR "177Lu lutetium" OR "177Lu" OR "177 Lu") AND (("dotatoc" OR "dota-toc" OR "DOTA-Phe1-Tyr3-octreotide" OR "DOTA Phe1 Tyr3 octreotide") OR ("edotreotide")) OR ("lutetium Lu 177 edotreotide" OR "Lu 177 edotreotide" OR "177Lu edotreotide" OR "177 Lu edotreotide" OR "Lu-DOTATOC" OR "177Lu-DOTATOC" OR "[177Lu]Lu-DOTA-TOC") |

Abbreviation: ENETS, European Neuroendocrine Tumor Society.

^a^In PubMed, various additional synonyms were checked that did not generate further hits: (177)Lu-DOTATOC OR (177LUDOTA)-TOC OR edotreotide lutetium LU-1772 OR Lutetium lu177 edotreotide OR DOTATOC Lu-177 OR (177Ludota)-toc OR Edotreotide lutetium Lu-177 OR RGO812Q0C8 OR Lutetium lu177 edotreotide [USAN] OR Lutetium Lu-177 DOTA-Tyr3-octreotide OR UNII-RGO812Q0C8 OR Lutetium Lu 177-edotreotide OR Lutetium Lu-177-edotreotide OR WHO 12604 OR 321835-55-6 OR LUTETIUM LU-177 EDOTREOTIDE OR LUTETIUM (177LU) EDOTREOTIDE OR LUTETIUM (177LU) EDOTREOTIDE [WHO-DD] OR (S2,S7-cyclo[[4,7,10-tris(carboxylato-kO-methyl)-1,4,7,10-tetraaza-k3N4,N7,N10-cyclodec-1-yl]acetyl-kO]-D-phenylalanyl-L-cysteinyl-L-tyrosyl-D-tryptophyl-L-lysyl-L-threonyl-L-cysteinyl-L-threoninol)lutetium[177Lu](3-) OR [N2.1-([4,7,10-tris[(carboxylato-kappaO)methyl]-1,4,7,10-tetraazacyclododecan-1-yl-kappa4N1,N4,N7,N10]acetyl-kappaO)- S3.2,S3.7-cyclo(D-phenylalanyl-L-cysteinyl-L-tyrosyl-D- tryptophyl-L-lysyl-L-threonyl-L-cysteinyl-L- threoninol)](177Lu)lutetium OR Lutetium-177Lu, [N-[2-[4,7,10-tris[(carboxy-O)methyl]-1,4,7,10-tetraazacyclododec-1-yl]acetyl-O]-D-phenylalanyl-L-cysteinyl-L-tyrosyl-D-tryptophyl-L-lysyl-L-threonyl-N-[(1R,2R)-2-hydroxy-1-(hydroxymethyl)propyl]-L-cysteinamide cyclic (2-->7)-disulfidato(3-)]).

**SUPPLEMENTAL TABLE 3** Newcastle-Ottawa scale checklist for cohort studies.

| **Selection** |
| --- |
| 1) Representativeness of the exposed cohort  a) truly representative of the average _______________ (describe) in the community  b) somewhat representative of the average ______________ in the community  c) selected group of users (e.g., nurses, volunteers)  d) no description of the derivation of the cohort |
| 2) Selection of the non-exposed cohort  a) drawn from the same community as the exposed cohort  b) drawn from a different source  c) no description of the derivation of the non-exposed cohort |
| 3) Ascertainment of exposure  a) secure records (e.g., surgical records)  b) structured interview  c) written self-report  d) no description |
| 4) Demonstration that outcome of interest was not present at start of study  a) yes  b) no |
| **Comparability** |
| 1) Comparability of cohorts based on the design or analysis  a) study controls for _____________ (select the most important factor)  b) study controls for any additional factor (this criterion could be modified to indicate specific control for a second important factor) |
| **Outcome** |
| 1) Assessment of outcome  a) independent blind assessment  b) record linkage  c) self-report  d) no description |
| 2) Was follow-up long enough for outcomes to occur?  a) yes (select an adequate follow-up period for the outcome of interest)  b) no |
| 3) Adequacy of follow-up of cohorts  a) complete follow-up—all subjects accounted for  b) subjects lost to follow-up unlikely to introduce bias - small number lost - > ____ % (select an adequate %) follow-up, or description provided of those lost)  c) follow-up rate < ____% (select an adequate %) and no description of those lost  d) no statement |

*Note:* A study can be awarded a maximum of one star for each numbered item within the Selection and Outcome categories. A maximum of two stars can be given for Comparability.

**SUPPLEMENTAL TABLE 4** Critical appraisal of included studies.

| **Publication, author year** | **Selection domain** | **Comparability domain** | **Outcome/ exposure domain** | **Total score** | **Quality*** |
| --- | --- | --- | --- | --- | --- |
| **Forrer et al. 2005^1^** | 2 | 0 | 3 | 5 | Poor |
| **Theiler et al. 2021^2^** | 4 | 2 | 3 | 9 | Good |
| **Kobayashi et al. 2021^3^** | 4 | 0 | 3 | 7 | Poor |
| **Radojewski et al. 2015^4^** | 3 | 2 | 3 | 8 | Good |
| **Ruhwedel et al. 2021^5^** | 4 | 1 | 2 | 7 | Good |
| **Baum et al. 2016^6,7^** | 3 | 0 | 3 | 6 | Good |
| **Luna-Gutiérrez et al. 2023^8^** | 3 | 0 | 3 | 6 | Good |
| **Laudicella et al. 2022^9^** | 4 | 0 | 3 | 5 | Poor |

*Good Quality: a score of 3 or 4 in the Selection domain, a score of 1 or 2 in the Comparability domain, and a score of 2 or 3 in the Outcome/Exposure domain; Fair Quality: a score of 2 in the Selection domain, a score of 1 or 2 in the Comparability domain, and a score of 2 or 3 in the Outcome/Exposure domain; Poor Quality: a score of 0 or 1 in the Selection domain, or a score of 0 in the Comparability domain, or a score of 0 or 1 in the Outcome/Exposure domain.

**SUPPLEMENTAL TABLE 5** Study inclusion criteria and assessments.

|  | **Forrer et al. 2005^1^** | **Theiler et al. 2021^2^** | **Kobayashi  et al. 2021^3^** | **Radojewski  et al. 2015^4^** | **Ruhwedel  et al. 2021^5^** | **Baum  et al. 2016^6,7^** | **Luna-Gutiérrez  et al. 2023^8^** | **Laudicella  et al. 2022^9^** |
| --- | --- | --- | --- | --- | --- | --- | --- | --- |
| Confirmed NET diagnosis | ⚫ | ⚫ | ⚫ | ⚫ | ⚫ | ⚫ | ⚫ | ⚫ |
| Advanced or Stage IVc disease or presence of distant metastases | ⚫^a^ | ⚫ | ⚫^a^ | ⚫ | ⚫ | ⚫ | ⚫ | ⚫ |
| Progressive NETs | ⚫ | ⚫ |  |  | ⚫ | ⚫ | ⚫ |  |
| Adequate bone marrow, renal, and hepatic function | ⚫ | ⚫ | ⚫ | ⚫ | ⚫ | ⚫^b^ | ⚫ | ⚫^c^ |
| Confirmation of SSTR expression or tumor uptake via functional imaging (PET/CT or scintigraphy) |  | ⚫ | ⚫ | ⚫ | ⚫ | ⚫^d^ | ⚫ | ⚫ |
| Visible tumor uptake on pretherapy SSTR2 scintigraphy |  |  | ⚫ | ⚫ |  |  |  | ⚫ |
| CgA assessed before each RPT cycle and at end of treatment |  |  |  |  | ⚫ | ⚫ |  | ⚫ |

Abbreviations: CgA, chromogranin A; CT, computed tomography; eGFR, estimated glomerular filtration rate; NET, neuroendocrine tumor; PET, positron emission tomography; RPT, radiopharmaceutical therapy; SSTR, somatostatin receptor.

^a^All patients had advanced/metastatic NETs, but this was not a study inclusion criterion. ^b^Not a specified inclusion criterion but measured before each RPT cycle (eGFR was measured in 32 patients, 34% of whom had grade 2 renal impairment at baseline). ^c^Adequate hepatic function not specified. ^d^Information taken from Baum et al. 2018.^7^

**SUPPLEMENTAL TABLE 6** Data sources, linked publications, and analysis populations for the meta-analysis.

| **Center location** | **Primary/original source publication** | ***N*** | **Linked publications** | **Data source used in meta-analysis of efficacy** | **Available analysis population(s)** |
| --- | --- | --- | --- | --- | --- |
| Basel, Switzerland | Forrer et al. 2005^1^ | 27 | - | Forrer et al. 2005^1^ | All-NETs (overall) |
| Basel, Switzerland | Theiler et al. 2021^2^ | 51 | - | Updated data provided by Dr. Nicolas | All-NETs (overall) GEP-NETs (subgroup) |
| Basel, Switzerland | Kobayashi et al. 2021^3^ | 19 | ^10^ | Kobayashi et al. 2021^3^ | All-NETs (overall) |
| Basel, Switzerland | Radojewski et al. 2015^4^ | 141 | ^11,12^ | Updated data provided by Prof. Walter | All-NETs (overall) |
| Berlin, Germany | Ruhwedel et al. 2021^5^ | 141 | ^13-17^ | Updated data provided by Dr. Ruhwedel/Ruhwedel et al. 2025^17^ (additional baseline characteristics data taken from^16,17^) | All-NETs (overall) GEP-NETs (subgroup) |
| Bad Berka, Germany | Baum et al. 2016^6^ | 56 | ^7,18-23^ | Baum et al. 2016^6^ (additional study information taken from^7^) | All-NETs (overall) GEP-NETs (subgroup) |
| Italy | Laudicella et al. 2022^9^ | 38 | - | Laudicella et al 2022^9^ (and updated data provided by Dr. Laudicella) | GEP-NETs (only) |
| Mexico | Luna-Gutiérrez et al. 2023^8^ | 187 | - | Luna-Gutiérrez et al. 2023^8^ | All-NETs (overall) GEP-NETs (subgroup) |

Abbreviation: (GEP-)NET, (gastro-enteropancreatic) neuroendocrine tumor.

**SUPPLEMENTAL TABLE 7** Sensitivity analyses of key efficacy outcomes.

| **Outcome (population)** | **Scenario** |  | **Proportion [95% CI]** | |
| --- | --- | --- | --- | --- |
|  |  | **Heterogeneity I^2^ [*p*-value]** | **Random-effect model** | **Fixed-effect model** |
| ORR  (GEP-NETs) | Double-arcsine transformation | 90% [*< .*01] | 0.34 [0.17*–*0.54] | 0.28 [0.22*–*0.34] |
|  | Logit transformation | 88% [*< .*01] | 0.34 [0.17*–*0.55] | 0.33 [0.26*–*0.40] |
|  | Log transformation | 87% [*< .*01] | 0.32 [0.18*–*0.59] | 0.38 [0.32*–*0.46] |
| ORR  (All-NETs) | Double-arcsine transformation | 89% [*< .*01] | 0.19 [0.08*–*0.32] | 0.14 [0.11*–*0.18] |
|  | Logit transformation | 87% [*< .*01] | 0.18 [0.09*–*0.33] | 0.20 [0.16*–*0.25] |
|  | Log transformation | 87% [*< .*01] | 0.17 [0.09*–*0.34] | 0.24 [0.19*–*0.29] |
| DCR (GEP-NETs) | Double-arcsine transformation | 85% [*< .*01] | 0.78 [0.60*–*0.92] | 0.76 [0.70*–*0.82] |
|  | Logit transformation | 64% [= .04] | 0.79 [0.59*–*0.91] | 0.75 [0.69*–*0.80] |
|  | Log transformation | 92% [*< .*01] | 0.77 [0.63*–*0.93] | 0.90 [0.86*–*0.94] |
| DCR (All-NETs) | Double-arcsine transformation | 97% [*< .*01] | 0.57 [0.33*–*0.79] | 0.50 [0.45*–*0.55] |
|  | Logit transformation | 95% [*< .*01] | 0.57 [0.33*–*0.78] | 0.51 [0.46*–*0.56] |
|  | Log transformation | 94% [*< .*01] | 0.49 [0.28*–*0.87] | 0.74 [0.69*–*0.79] |
| All-cause mortality (GEP-NETs) | Double-arcsine transformation | 92% [*< .*01] | 0.39 [0.18*–*0.62] | 0.44 [0.39*–*0.50] |
|  | Logit transformation | 86% [*< .*01] | 0.38 [0.18*–*0.64] | 0.49 [0.43*–*0.56] |
|  | Log transformation | 83% [*< .*01] | 0.38 [0.22*–*0.67] | 0.54 [0.49*–*0.61] |
| All-cause mortality  (All-NETs) | Double-arcsine transformation | 85% [*< .*01] | 0.48 [0.37*–*0.60] | 0.49 [0.45*–*0.54] |
|  | Logit transformation | 84% [*< .*01] | 0.48 [0.37*–*0.60] | 0.49 [0.45*–*0.54] |
|  | Log transformation | 84% [*< .*01] | 0.48 [0.38*–*0.60] | 0.54 [0.50*–*0.59] |

Abbreviations: DCR, disease control rate; (GEP-)NET, (gastro-enteropancreatic) neuroendocrine tumor; ORR, objective response rate.

**SUPPLEMENTAL FIGURE 1** Meta-analysis of disease control rates in the (A) GEP-NET and (B) All-NET populations.^
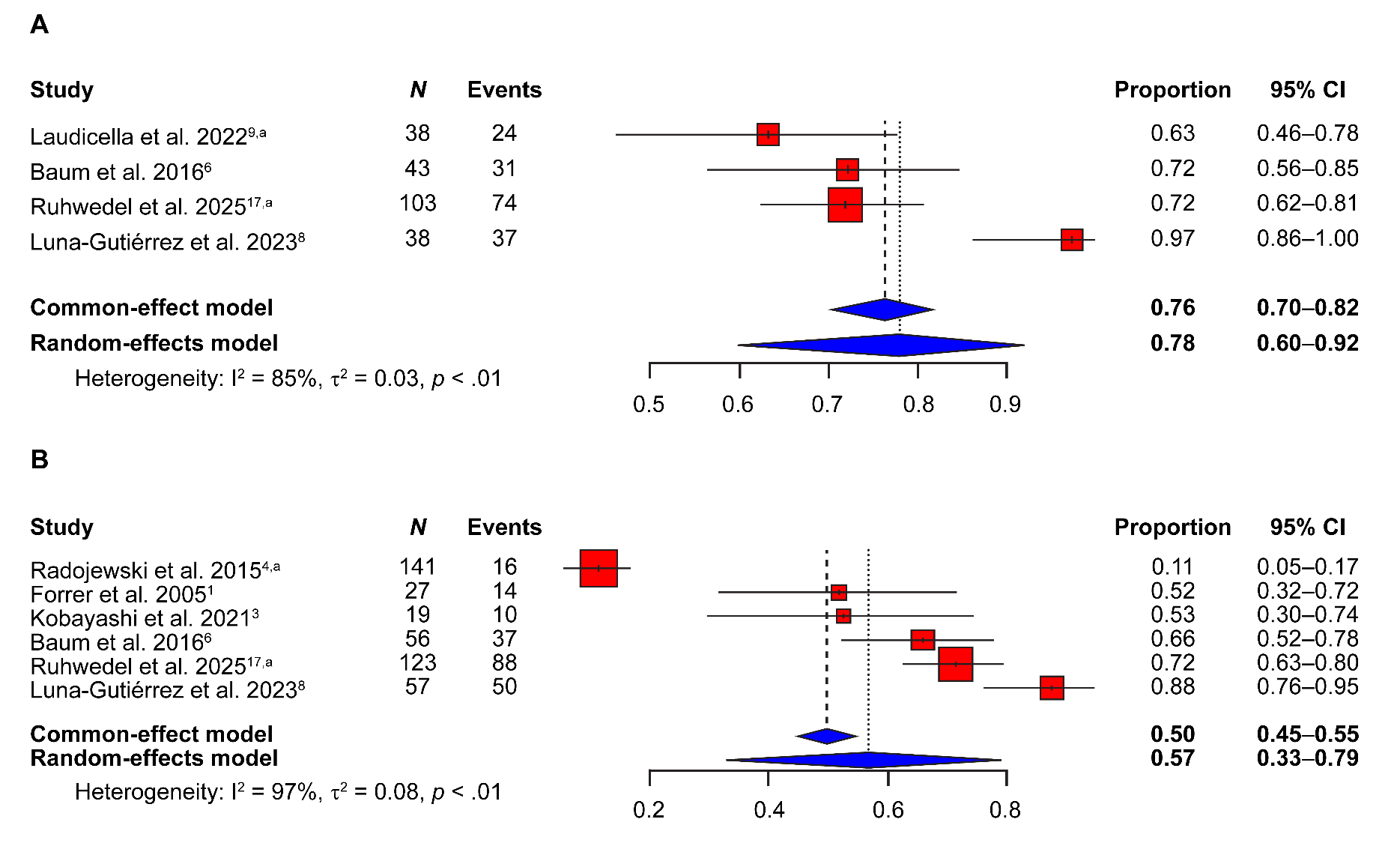
^

Abbreviation: (GEP-)NET, (gastro-enteropancreatic) neuroendocrine tumor.

^a^Updated and/or additional unpublished data provided by the investigator of this study.

# References

1. Forrer F, Uusijärvi H, Storch D, Maecke HR, Mueller-Brand J. Treatment with 177Lu-DOTATOC of patients with relapse of neuroendocrine tumors after treatment with 90Y-DOTATOC. *J Nucl Med*. 2005;46(8):1310-1316.

2. Theiler D, Cattaneo M, Dierickx LO, et al. Safety and efficacy of peptide-receptor radionuclide therapy in elderly neuroendocrine tumor patients. *Cancers (Basel)*. 2021;13(24):6290. doi:10.3390/cancers13246290.

3. Kobayashi N, Wild D, Kaul F, et al. Retrospective study of peptide receptor radionuclide therapy for Japanese patients with advanced neuroendocrine tumors. *J Hepatobiliary Pancreat Sci*. 2021;28(9):727-739. doi:10.1002/jhbp.1014.

4. Radojewski P, Dumont R, Marincek N, et al. Towards tailored radiopeptide therapy. *Eur J Nucl Med Mol Imaging*. 2015;42(8):1231-1237. doi:10.1007/s00259-015-3030-9.

5. Ruhwedel T, Rogasch JMM, Huang K, et al. The prognostic value of the De Ritis ratio for progression-free survival in patients with NET undergoing [(177)Lu]Lu-DOTATOC-PRRT: a retrospective analysis. *Cancers (Basel)*. 2021;13(4):635. doi:10.3390/cancers13040635.

6. Baum RP, Kluge AW, Kulkarni H, et al. [(177)Lu-DOTA](0)-D-Phe(1)-Tyr(3)-Octreotide ((177)Lu-DOTATOC) for peptide receptor radiotherapy in patients with advanced neuroendocrine tumours: a phase-II study. *Theranostics*. 2016;6(4):501-510. doi:10.7150/thno.13702.

7. Baum RP, Kulkarni HR, Singh A, et al. Results and adverse events of personalized peptide receptor radionuclide therapy with (90)Yttrium and (177)Lutetium in 1048 patients with neuroendocrine neoplasms. *Oncotarget*. 2018;9(24):16932-16950. doi:10.18632/oncotarget.24524.

8. Luna-Gutiérrez M, Hernández-Ramírez R, Soto-Abundiz A, et al. Improving overall survival and quality of life in patients with prostate cancer and neuroendocrine tumors using (177)Lu-iPSMA and (177)Lu-DOTATOC: experience after 905 treatment doses. *Pharmaceutics*. 2023;15(7):1988. doi:10.3390/pharmaceutics15071988.

9. Laudicella R, Comelli A, Liberini V, et al. [(68)Ga]DOTATOC PET/CT radiomics to predict the response in GEP-NETs undergoing [(177)Lu]DOTATOC PRRT: the "theragnomics" concept. *Cancers (Basel)*. 2022;14(4):984. doi:10.3390/cancers14040984.

10. Hasegawa S, Kobayashi N, Wild D, et al. Factors contributing to tumor shrinkage after peptide receptor radionuclide therapy in patients with unresectable neuroendocrine tumors. *Cancers (Basel)*. 2022;14(14). doi:10.3390/cancers14143317.

11. Romer A, Seiler D, Marincek N, et al. Somatostatin-based radiopeptide therapy with [177Lu-DOTA]-TOC versus [90Y-DOTA]-TOC in neuroendocrine tumours. *Eur J Nucl Med Mol Imaging*. 2014;41(2):214-222. doi:10.1007/s00259-013-2559-8.

12. Umlauft M, Radojewski P, Spanjol PM, et al. Diabetes mellitus and its effects on all-cause mortality after radiopeptide therapy for neuroendocrine tumors. *J Nucl Med*. 2017;58(1):97-102. doi:10.2967/jnumed.116.180687.

13. Galler M, Rogasch J, Buch F, Bluemel S, Schatka I, Amthauer H. Quantification in Lutetium-177-DOTATOC SPECT/CT using NaI detectors: application to neuroendocrine tumor patients. *NuklearMedizin*. 2020;59(2):165.

14. Galler M, Rogasch JMM, Huang K, et al. Prognostic value of the largest lesion size for progression-free survival in patients with NET undergoing salvage PRRT with [(177)Lu]Lu-DOTATOC. *Cancers (Basel)*. 2022;14(7):1768. doi:10.3390/cancers14071768.

15. Mogl MT, Dobrindt EM, Buschermöhle J, et al. Influence of gender on therapy and outcome of neuroendocrine tumors of gastroenteropancreatic origin: a single-center analysis. *Visc Med*. 2020;36(1):20-27. doi:10.1159/000505500.

16. Wetz C, Ruhwedel T, Schatka I, et al. Plasma markers for therapy response monitoring in patients with neuroendocrine tumors undergoing peptide receptor radionuclide therapy. *Cancers (Basel)*. 2023;15(24). doi:10.3390/cancers15245717.

17. Ruhwedel T, Rogasch J, Schatka I, et al. Beyond similarities: overall survival and prognostic insights from [¹⁷⁷Lu]Lu-DOTATOC therapy in neuroendocrine tumors. *Eur J Nucl Med Mol Imaging*. 2025. doi:10.1007/s00259-025-07221-2.

18. Hörsch D, Ezziddin S, Haug A, et al. Effectiveness and side-effects of peptide receptor radionuclide therapy for neuroendocrine neoplasms in Germany: a multi-institutional registry study with prospective follow-up. *Eur J Cancer*. 2016;58:41-51. doi:10.1016/j.ejca.2016.01.009.

19. Aalbersberg EA, Huizing DMV, Walraven I, et al. Parameters to predict progression-free and overall survival after peptide receptor radionuclide therapy: a multivariate analysis in 782 patients. *J Nucl Med*. 2019;60(9):1259-1265. doi:10.2967/jnumed.118.224386.

20. Zhang J, Kulkarni HR, Singh A, Niepsch K, Müller D, Baum RP. Peptide receptor radionuclide therapy in grade 3 neuroendocrine neoplasms: safety and survival analysis in 69 patients. *J Nucl Med*. 2019;60(3):377-385. doi:10.2967/jnumed.118.215848.

21. Graf J, Pape UF, Jann H, et al. Prognostic significance of somatostatin receptor heterogeneity in progressive neuroendocrine tumor treated with Lu-177 DOTATOC or Lu-177 DOTATATE. *Eur J Nucl Med Mol Imaging*. 2020;47(4):881-894. doi:10.1007/s00259-019-04439-9.

22. Zhang J, Liu Q, Singh A, Schuchardt C, Kulkarni HR, Baum RP. Prognostic value of (18)F-FDG PET/CT in a large cohort of patients with advanced metastatic neuroendocrine neoplasms treated with peptide receptor radionuclide therapy. *J Nucl Med*. 2020;61(11):1560-1569. doi:10.2967/jnumed.119.241414.

23. Zhang J, Jakobsson V, Chen X, Baum RP. 177Lu/90Y-SSTR peptide receptor radionuclide therapy of neuroendocrine tumor cardiac metastases. *J Nucl Med*. 2023;64(Suppl 1):P1607.
